# Distribution of enteroviruses in hospitalized children with hand, foot and mouth disease and relationship between pathogens and nervous system complications

**DOI:** 10.1186/1743-422X-9-8

**Published:** 2012-01-09

**Authors:** Wei Xu, Chun-feng Liu, Li Yan, Jiu-jun Li, Li-jie Wang, Ying Qi, Rui-bo Cheng, Xiao-yu Xiong

**Affiliations:** 1 Department of Pediatrics, Shengjing Hospital of China Medical University, Shenyang 110817, China; 2 Department of Prevention, Shengjing Hospital of China Medical University, Shenyang 110817, China; 3 Virus laboratory, Shengjing Hospital of China Medical University, Shenyang 110817, China; 4 Department of Preventive Dentistry, School of Stomatology, China Medical University, Shenyang 110002, China

**Keywords:** EV71, CA16, HFMD, Pathogen, Children, Nervous system complication

## Abstract

**Background:**

To explore the relationship between enteroviruses and hospitalized children with hand, foot and mouth disease (HFMD) complicated with nervous system disease. 234 hospitalized HFMD patients treated in Shengjing Hospital, Liaoning Province were analyzed retrospectively. Based on the presence and severity of nervous system disease, the patients were grouped as follows: general patients, severely ill patients, critically ill patients and fatal patients. Based on the detected pathogen, the patients were grouped as follows: Enterovirus 71 (EV71) infection, coxsackie A16 (CA16) infection and other enterovirus (OE) infection.

**Results:**

Of the 423 hospitalized patients, most were admitted in July 2010(129/423, 30.5%). Enteroviruses were detected in 177(41.8%). 272/423 patients were male (64.3%), and fatal patients had the greatest proportion of male patients (*p *< 0.05). EV71 infection was found in 89/423 patients (21%). CA16 infection was detected in 8/423 patients (16.1%). Compared to group CA16, patients in group EV71 were hospitalized earlier, and the duration of hospitalization was longer (*p *< 0.05). Of the 92 patients with nervous system damage, 65 were infected with EV71 and 19 were infected with CA16. Among these CA16 infected patients, 2 had brainstem encephalitis and 1 had AFP. There were more patients with nervous system dysfunction in group EV71 than in groups CA16 or OE (*p *< 0.05). The 5 fatalities all occurred in group EV71 patients (*p *< 0.05). Infection with EV71 was most likely to cause neurogenic pulmonary edema (*p *< 0.05). Patients in group EV71 had a higher rate of suffering from coma and limb movement disorder than patients in groups CA16 or OE (*p *< 0.05).

**Conclusion:**

The disease progresses faster in EV71-infected HFMD patients. These patients are more likely to suffer nervous system damage, neurogenic pulmonary edema, severe sequelae or death. CA16 and other enteroviruses can also cause HFMD with severe nervous system complications.

## Background

Since first reported by Robin Son in 1958 [1], numerous widespread outbreaks of hand, foot and mouth disease (HFMD) have occurred in eastern and southeastern Asia, including Singapore [2], South Korea [3], Malaysia [4], Japan [5], Vietnam [6], mainland China [7,8] and Taiwan [9,10]. HFMD was first reported in mainland China in 1981; since then it has prevailed in most provinces of China. Nationwide HFMD outbreaks have occurred in China since 2008, with most of the cases affecting children ≤ 5 years of age [11]. After this event HFMD has been made a nationally notifiable disease. Despite nationwide effort, 1,795,336 cases were diagnosed annually in 2010 with 905 deaths. HFDM is a common infectious disease that can be caused by more than 20 different enteroviruses, and the symptoms are generally mild and self-limited. Its main manifestations are fever, rash and ulcers in areas such as the oral mucosa and the hands, feet and buttocks. A small proportion of children patients can experience severe complications, including encephalitis, pneumonia, myocarditis, brain-stem encephalitis and acute flaccid paralysis (AFP). High mortality and severe sequelae can be anticipated when the disease is complicated by neurogenic pulmonary edema, rapid disease progression [12,13]. HFMD has a disease burden as severe as that imposed by any other pediatric infectious disease and has already received considerable attention from all health sectors at both national and local levels.

Unlike most infectious diseases, it is very difficult to distinguish between the possible pathogens (Enterovirus 71 and Coxsackie A16) in a clinical context, possibly because EV71 and CA16 are closely related. Enterovirus 71 (EV71) and coxsackie A16 (CA16) are small, non-enveloped, positive-stranded RNA viruses that belong to human enterovirus species A, genus *Enterovirus*, family Picornaviridae. Human EV71 and CA16 are two major pathogens of HFMD in children. These viruses can also cause many other diseases, many of which manifest as herpes angina or flu-like symptoms [14]. Together with CA7 and CA14, these viruses form a distinct genetic subgroup within cluster A of the genus *Enterovirus *[15]. Recent studies reported that severe nervous system damage happened more in patients infected with EV71. Some reports further suggest that despite the close genetic relationship between EV71 and CA16, only EV71 has the potential to cause neurological disease in acute infection [16,17]; however, we disagree with this statement. This report summarizes the clinical manifestations of disease and the distribution of pathogens in HFMD patients, based on patients whose cases were complicated by nervous system disease.

## Results

In 2010, our hospital treated a total of 6,027 cases of pediatric HFMD. Among these, 423 patients (7.0%) were hospitalized. Hospital stay lasted from 1 to 101 days (median 9 days). 78 patients were referred to PICU with the stay in PICU lasting 1 to 101 days (median 18 days). The overall patient ages ranged from 3 months to 12.5 years, with an average age of 2.71 years.

### Demographic characteristics and clinical manifestations

**Figure 1:**
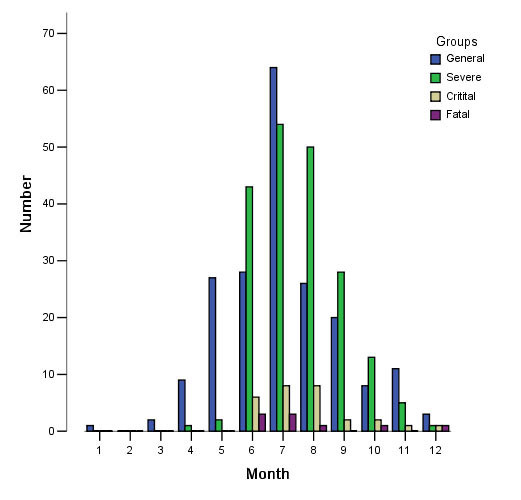
**Distribution of HFMD patients in different months**.

The 423 patients hospitalized with HFMD were mainly admitted between May and September. Within that period, most patients (129/423, 30.5%) were admitted in July. August and June followed, with 85/423 (20.1%) and 80/423 (18.9%), respectively. Among all admitted patients, 189/423 (44.7%) were general patients, 197/423 (46.6%) had severe clinical manifestations, 28/423 (6.6%) were critically ill and 9/423 (2.1%) died. The severely ill, critically ill and fatal cases were most frequently seen in June, July and August. July had the greatest number of severe cases (54/197, 27.4%). The number of critically ill patients was equally distributed between July and August (7/28, 25%). An equal number of fatalities occurred in June and July (3/9, 33.3%). A detailed distribution is shown in Figure 1.

The examination of throat swab and/or stool specimens from these 423 patients revealed the presence of a viral pathogen in 177 cases (41.84%). Among the 177 patients, there were 85 general patients, 71 severely ill patients, 16 critically ill patients and 5 deaths. There was no significant difference in the pathogen detection rate across groups (*p *> 0.05). The overall patient ages ranged from 3 months to 12.5 years, with an average age of 2.71 years. There were significant differences in the average ages among the 4 groups (*p *< 0.05). Among general, severely ill and critically ill groups, patients who had experienced more severe symptoms had more little ages, whereas the group of patients who died showed less significant difference in age compared to the other three groups. Male preferences were detected in the group with severe disease. The proportion of patients from rural areas was 268/423 (63.3%), but regional differences were not apparent among severe cases (*p *> 0.05). Patients in the group with general disease were hospitalized later than those in other 3 group t (*p *< 0.05). The time for patients in the group with critically disease stayed in hospital was the longest (*p *< 0.05). The rate of critically group in term of the number of patients with white blood cell counts > 12 × 10^9 ^in the initial regular blood test was the highest. Meanwhile patients with critically disease suffered fever for longer too. In total, 89/423 (21%) EV71 cases were detected, with a higher detection rate in patients with more severe manifestations. A total of 68/423 (16.1%) CA16 cases were detected, with a higher detection rate in patients less severely affected. Other enteroviruses (20/423, 4.7%) were also detected, with no significant difference in distribution among the 4 groups. Patients who had experienced more severe symptoms ended up with worse outcome (*p *< 0.05). A detailed distribution of the neurological sequelae is provided in Table 1.

**Table 1:** Analysis of the clinical data for 423 hospitalized patients

	general	severely	critically	fatal	*p *value	Total
**Case number (n)**	189	197	28	9		**423**
**Number of cases in which a pathogen was detected (n)**	85(45.0%)	71(36%)	16(57.1%)	5(55.6%)	**0.076**	**177(41.8%)**
Age*(Years)^①^	3.045 ± 2.102	2.546 ± 1.730	1.893 ± 1.397	2.056 ± 1.509	**0.003**	
**Gender(male, n)**	125(66.1%)	114(57.9%)	25(89.3%)	8(88.9%)	**0.003**	**272(64.3%)**
**Region(from countryside, n)**	116(61.4%)	124(62.9%)	23(82.1%)	5(55.6%)	**0.185**	**268(63.4%)**
**Time from onset to hospitalization(d)**^①^	4.44 ± 1.37	3.89 ± 1.26	3.92 ± 1.02	3.81 ± 0.96	**0.000**	
**Duration of hospitalization(d)**^②^	8.85 ± 2.46	11.63 ± 5.04	49.51 ± 33.20	4.55 ± 1.91	**0.000**	
**WBC > 12 × 10^9 ^(n)**	86(45.5%)	121(61.4%)	27(96.4%)	8(88.9%)	**0.000**	**242(57.2%)**
**Duration of initial fever(d)**^②^	5.24 ± 1.54	6.33 ± 1.94	8.38 ± 6.42	6.42 ± 2.25	**0.012**	
**Highest fever temperature (°C)**^③^	38.76 ± 0.46	38.846 ± 0.62	39.206 ± 0.51	39.07 ± 0.41	**0.032**	
**Hand or foot rash (n)**	173(91.5%)	183(92.9%)	23(82.1%)	6(66.7%)	**0.017**	**385(91.0%)**
**Oral rash (n)**	121(64.0%)	118(59.9)	17(74.6)	4(44.4%)	**0.608**	**260(61.5%)**
**Buttock rash (n)**	76(40.2%)	81(41.1%)	6(21.4%)	2(22.2%)	**0.159**	**165(39.0)**
**No rash (n)**			2(7.1%)	1(11.1%)	**0.000**	**3(0.7%)**
**EV71 (n)**	24(12.7%)	47(23.9%)	13(46.4%)	5(55.6%)	**0.000**	**89(21%)**
**CA16 (n)**	49(25.9%)	18(9.1%)	1^©^(3.6%)	0	**0.000**	**68(16.1%)**
**Other enteroviruses (n)**	12(6.3%)	6(3%)	2(7.1%)	0	**0.369**	**20(4.7%)**
**Patients with neurological dysfunction upon discharge (n)**	0	2^®^(1%)	11(39.3%)		**0.000**	**13(3.1%)**

①There is statistically significant difference between general group and the other 3 groups but no difference in comparison to the other 3 groups. ②There is statistically significant difference between all groups. ③There is no difference between general groups and severely group or critically group and fatal group, but there is significant difference between each one of the former and the latter two groups. # There was no repeat counting in calculating the distribution of disease types. When neurogenic pulmonary edema or shock and brain stem encephalitis coexisted in the same patient, the former was counted. When neurogenic shock and pulmonary edema coexisted, pulmonary edema was counted. §The denominator of the fraction represents the cases examined, whereas the numerator represents the number of patients with this abnormality. ¤Among the 5 fatal cases, 2 died of neurogenic pulmonary edema, 2 died of shock and 1 patient was declared brain-dead

### Data characteristics of the clinical distribution of the enteroviruses

**Figure 2:**
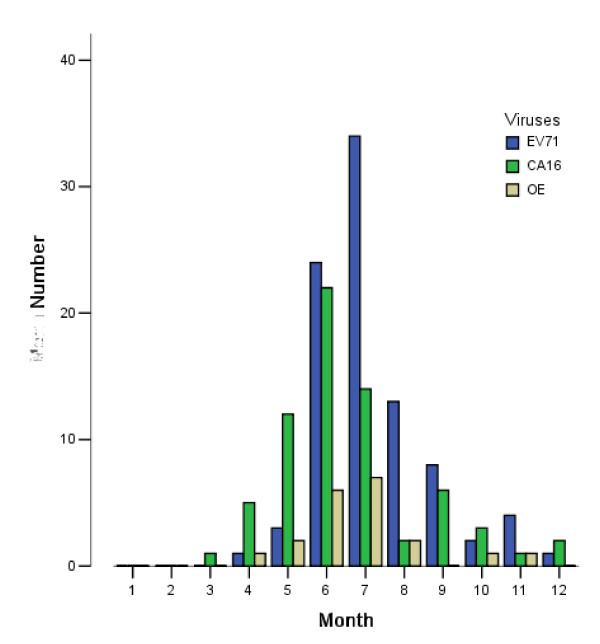
**Pathogen distributions in different months**.

Samples from the 423 hospitalized HFMD patients were examined, and 177(41.8%) tested positive for enterovirus. The results are shown in Figure 2.

Patients with EV71 infection were hospitalized earlier and for longer periods than those with CA16 infection. The difference was significant (*p *< 0.05). Patients with EV71 infection had the highest average temperature (39.1°C), which was significantly different from the average temperatures in the other 2 groups (*p *< 0.05). Three critically ill patients had no rash. HFMD was confirmed in all patients with EV71 infection, although there was no significant difference compared to the other 2 groups (*p *> 0.05). EV71, CA16 and other enteroviruses are all capable of causing nervous system damage and leading to a certain degree of neurologic dysfunction. There were 92 patients (52%) with nervous system damage; the patients with EV71 were especially at risk (73%). The incidence in the CA16 group was 19 (27.9%), and the incidence in patients infected with other enteroviruses was 1 (5%); the differences among the 3 groups were significant (*p *< 0.05). Among the 13 patients with some neurologic dysfunction at discharge, 11 were infected with EV71, which was significantly different from the rates found in the other 2 groups (*p *< 0.05). The 13 patients with neurologic dysfunction after treatment in the acute phase included the following: 5 patients with difficulty swallowing or coughing, a tendency to cough, a tendency to salivate and difficulty in getting rid of sputum; 5 patients with limb movement dysfunction, 2 of whom had grade-III muscle strength in both lower extremities and 3 of whom had grade-III muscle strength in a single limb and 1 patient with mental decline. After 3 months of mechanical ventilation in the pediatric intensive care unit (PICU), these patients showed stable vital signs and the presence of brain function; however, they still did not have regular, effective, spontaneous respiration. Two of these patients required prolonged mechanical ventilation and were transferred to local hospitals for further treatment. The 5 patients who died were infected with EV71; the difference in pathogen prevalence was significant (*p *< 0.05), as shown in Table 2.

**Table 2:** Comparison of the clinical data from patients with different enteroviruses

	EV71	CA16	OE	*p *value	Total
**Total (n)**	89	68	20		**177**
**Gender (Male, n)**	52(58.4%)	38(55.9%)	14(70%)	**0.528**	**104(58.8%)**
Age*(Years)	2.382 ± 2.102	2.588 ± 1.860	1.875 ± 1.146	**0.347**	
**Region (from countryside, n)**	57(64%)	41(60.3%)	13(65%)	**0.868**	**111(62.3%)**
**Time from onset to hospitalization (d)**^②^	3.258 ± 1.176	4 ± 1.258	3.6 ± 1.353	**0.001**	
**Duration of hospitalization (d)**	14.337 ± 17.796	9.338 ± 3.366	8.1 ± 3.354	**0.182**	
**WBC > 12 × 10^9 ^(n)**	51(57.3%)	32(47.1%)	11(55%)	**0.437**	**94(530.1%)**
**CRP > 8 mg/dl (n)**	4(4.5%)	3(4.4%)	1(5%)	**0.994**	**8(4.5%)**
**Duration of initial fever(d)**	6.034 ± 3.131	5.706 ± 1.684	5.45 ± 1.468	**0.547**	
**Highest fever temperature (°C)**^③^	39.144 ± 0.563	38.919 ± 0.494	38.92 ± 0.618	**0.025**	
**Hand or foot rash (n)**	81(91%)	67(98.5%)	20(100%)	**0.057**	**168(94.9%)**
**Oral rash (n)**	58(65.2%)	50(73.5%)	15(75%)	**0.451**	**123(69.5%)**
**Buttock rash (n)**	39(43.8%)	28(41.2%)	6(30%)	**0.525**	**73(41.2%)**
**No rash (n)**	3(2.2%)	0	0	**0.221**	**3(1.7%)**
**Accompanied nervous system damage**	65(73%)	19(27.9%)	8(40%)	**0.000**	**92(52%)**
**Patients with dysfunction upon discharge (n)**	11(12.4%)	1(1.5%)	1(5%)	**0.030**	**13(7.3%)**
**Death (n)**	5(7.3%)	0	0	**0.030**	**5(2.8%)**

*Patients aged 0-12 months were counted as being 0.5 years of age; patients aged 1-2 years were counted as being 1.5 years of age; patients aged 2-3 years were counted as being 2.5 years of age, etc. ¤Among the 5 fatal cases, 2 patients died of neurogenic pulmonary edema, 2 died of shock, and 1 was diagnosed as being brain-dead. ② Statistically significant difference between groups A and B but no difference in comparison to the other 2 groups. ③There is statistically significant difference between group A and groups B and C but no difference in comparison to the other 2 groups

The pathogen distribution in the 92 cases of HFMD patients with nervous system damage is shown in Table 3. Among the 3 groups, there was a significant difference observed in the prevalence of neurogenic pulmonary edema (*p *< 0.05). EV71 was the most frequently detected agent in patients for all the diseases studied. Compared to CA16 or other enteroviruses, EV71 was most likely to cause all severe neurological diseases except for encephalitis. CA16 and other enteroviruses were the most common agents in encephalitis. EV71-infected patients had a much higher incidence of clinical coma or limb movement disorder than the other 2 groups; these differences were significant (*p *< 0.05). Although EV71 infection was more likely to cause abnormal numbers of white blood cells in cerebrospinal fluid (CSF), there was no significant difference among the 3 groups (*p *> 0.05). In contrast to the results in Table 2, there were no significant differences among the different viruses in the frequency of neurological damage leading to dysfunction upon discharge or death *p *> 0.05.

**Table 3:** Data analysis of the clinical neurological damage caused by different pathogens

	EV71	CA16	OE	*p *value	Total
**Total (n)**	65(70.7%)	19(20.7%)	8(8.7%)		**92**
**Encephalitis (n)^#^**	39(60%)	16(82.4%)	6(75%)	**0.125**	**61(66.3%)**
**Acute flaccid paralysis (n)^#^**	2(3%)	1(5.3%)	0	**0.772**	**3(3.3%)**
**Brainstem encephalitis (n)^#^**	8(12.3%)	2(1.1%)	0	**0.572**	**10(10.9%)**
**Neurogenic pulmonary edema (n)^#^**	11(16.9%)	0	1(12.5%)	**0.047**	**11(12%)**
**Neurologic shock (n)^#^**	5(7.7%)	0	1(12.5%)	**0.215**	**6(6.5%)**
**Limb shaking (n)**	51(78.5%)	15(78.9%)	6(75%)	**0.973**	**72(78.3%)**
**Convulsion (n)**	21(32.3%)	4(21.1%)	2(25%)	**0.613**	**27(29.3%)**
**Vomiting (n)**	42(64.6%)	16(84.2%)	7(87.5%)	**0.141**	**65(70.7%)**
**Fatigue (n)**	47(72.3%)	17(89.5%)	5(62.5%)	**0.219**	**69(75%)**
**Coma (n)**	24(36.9%)	2(10.5%)	1(12.5%)	**0.046**	**27(29.3%)**
**Limb movement disorder (n)**	34(52.3%)	3(15.8%)	2(25%)	**0.010**	**39(42.4%)**
**Urinary retention (n)**	31(47.7%)	6(31.6%)	2(25%)	**0.266**	**39(42.4%)**
**CSFWBC > 10 × 10^6^/L (n)^§^**	21/44(47.7%)	8/14(57.1%)	3/6(50%)	**0.828**	**25/64(39.1%)**
**CSFPro ≥ 0.5 g/L (n)^§^**	15/44(34.1%)	5/14(35.7%)	2/6(33.3%)	**0.992**	**22/64(34.4%)**
**Ambulatory EEG abnormality (n)^§^**	17/32(53.1%)	4/13(30.8%)	1/5(20%)	**0.205**	**22/50(44%)**
**Head MRI abnormality (n)^§^**	13/32(40.6%)	5/15(33.3%)	2/6(33.3%)	**0.685**	**20/53(37.7%)**
**Patients with dysfunction upon discharge (n)**	11(16.9%)	1(5.3%)	1(12.5%)	**0.435**	**13(14.1%)**
**Death (n)^¤^**	5(7.7%)	0	0	**0.333**	**5(5.4%)**

# There was no repeat counting in calculating the distribution of disease types. When neurogenic pulmonary edema or shock and brain stem encephalitis coexisted in the same patient, the former was counted. When neurogenic shock and pulmonary edema coexisted, pulmonary edema was counted. §The denominator of the fraction represents the cases examined, whereas the numerator represents the number of patients with this abnormality. ¤Among the 5 fatal cases, 2 died of neurogenic pulmonary edema, 2 died of shock and 1 patient was declared brain-dead

## Discussion

Enterovirus infection is prevalent worldwide and usually prevales in warm weather, especially in summer and autumn, when it is associated with an increase in central nervous system infection [18]. In our study, the first patients with severe neurological impairment were admitted in April, and additional patients followed in June, July and August. Cases peaked in August and decreased afterward [19,20]. This seasonal distribution coincides with the trend in recent 3 years observed in Liaoning Province. In southern China, in Guangdong and Shandong, the flow of patients with severe symptoms started in March and April, possibly because of the warmer southern climate. Similar to recent studies [21], males account for 64.3% of all patients in this study and are prone to severe symptoms. Although some studies reported that aged 4-9 years [22] or 5-10 years [23] may be the most susceptive period, we found, as in many studies [13] that patients under 5 years of age are at the highest risk, with most severe symptoms in the youngest patients. Rural preference is also observed, especially in patients with the most severe symptoms. We cautiously attribute this to the large rural population of China, as rural areas have poorer health care conditions and poorer environmental health; To make things worse, the water sanitation standard is low in rural China [24] (Table 1).

Among the 6,027 patients, 423 (7.0%) were hospitalized; the major pathogen in HFMD has been reported to be CA16, which generally causes mild and self-limited symptoms. The main pathogens in Beijing in 2009 were CA16 (49.4%) and EV71 (36.4%) [25]. In recent outbreaks, however, EV71 infection has been the major cause of HFMD [26,27]. This virus was also the major pathogen in this study. EV71 accounted for 85 infections (45.0%), and CA16 accounted for 71 (36%). In severely ill and critically ill patients, EV71 accounts for an even higher proportion. It was responsible for all the 5 deaths, although the case number is limited. This distribution is different from previous observations (Table 1), possibly because the studied population includes only hospitalized patients with relatively severe symptoms. EV71 caused neurologic damage in 65 patients, (70.7%), and CA16 was the pathogen in 19 patients (20.7%). It was possible that in Shenyang in 2010, EV71 was the major infective agent, but there is currently no official evidence to support this claim.

A different distribution of the gene in the population might explain why, in the past decade, EV71 has mainly caused disease in Taiwan, Malaysia, mainland China, Japan and other Asia-Pacific regions [28]. Taiwan researchers found that the histocompatibility leukocyte antigen (HLA)-A33 gene was correlated with susceptibility to virus infection and HLA-A2 was closely correlated with the occurrence of pulmonary edema. EV71 is neurotropic and can directly cause central neurological impairment; the virus replicates at the lesion site [29,30]. EV71 VP capsid proteins can bind to ornithine decarboxylase, gene-trap ankyrin repeats (GTARs) and other proteins expressed in neurons in the brain and cause cell injury [31]. Besides, it activates various cytokine pathways or bypasses signaling pathways to induce apoptosis or necrosis [32-35]. Studies have shown that in a mouse model, IgG antibodies generated during EV71 infection can pass the blood-brain barrier and cross-react with host brain tissue [36].

Other enteroviruses, such as ECHO7, ECHO14, the CVB group, CA6 and CA10, can all cause pediatric HFMD [37-39]. Other major enteroviruses in our studies include ECHO14, CVB and CVA10. In recent years, these viruses have shown an increasing trend as causes of HFMD; in particular, CVA6 and CVA 10 have recently emerged as pathogens of HFMD outbreaks in other countries, including Singapore and Finland [40,41] With the exception of ECHO14, the echovirus serotypes identified and the CVB group viruses, including CVB3, CVB4 and CVB5, have been previously associated with cases of HFMD [18,42].

The expression of the scavenger receptor class B, Member 2 (SCARB2) receptor protein can facilitate virus propagation in cells that normally are not susceptible to EV71 infection and also cause pathogenesis in these cells. CA16 can infect cells through the same pathway, but most other enteroviruses in group A do not depend on this ligand [43,44]. CA16 is generally associated with fewer neurologic complications, with the exception of infrequent cases of aseptic meningitis [45]. Neurological injuries caused by CA16 are relatively mild and deaths are especially rare. Therefore, the disease has attracted little attention [46,47]. However, we find that CA16 can also cause considerable neurological impairment. Although most affected patients had mild symptoms, three experienced severe, complicated nervous system injuries, two experienced brain-stem encephalitis and one suffered from AFP. The mechanism of pathogenesis, however, is not yet known, and it is not known whether neurons have receptors for EV71.

Neurogenic shock and neurogenic pulmonary edema occur simultaneously under most conditions and are highly dangerous; even if the circulation and lung function recover, brain and peripheral nervous function are more difficult to protect, and all of the 9 deaths in this study had neurogenic shock and/or neurogenic pulmonary edema. Death frequently occurs during the early stage of the disease (1st week), and brain death could occur even after circulation and respiratory functions are restored Even if the rescue is successful, the clinical recovery for these child patients is very slow. Some require prolonged mechanical ventilation to allow time for recovery; others require even longer ventilation to recover body movement and cranial nerve (glossopharyngeal nerve, vagus nerve or hypoglossal nerve) functions. Brainstem injury has long been considered a major cause of pulmonary edema [21,48]. In previous studies, however, pulmonary edema did not occur in monkeys with brainstem injury; therefore, other mechanisms may lead to EV71-related pulmonary edema [49]. Recent studies show that increased levels of the inflammatory cytokines interleukin-l (IL-1), interleukin-6 (IL-6), interleukin 10 (IL-10), interleukin 13 (IL- 13), interferon γ (IFN-γ) and tumor necrosis factor α (TNF-α) and immune suppression of CD3 and CD4 T-cells and natural killer (NK) cells are related to pulmonary edema [50]. The release of various inflammatory factors stimulates a radical inflammatory response and the release of catecholamines, causing an increased release of interleukin-8 (IL-8) and changes in pulmonary capillary permeability, leading to pulmonary edema [5,51,52].

The distribution of rash in HFMD patients infected by various viruses in this study showed no statistically significant differences, but three EV71-infected patients did not have any rash, and similar cases, as have been previously reported [3,53]. Convulsions, coma, body movement dysfunction and urinary retention mainly occur in EV71-infected patients because of their severe nervous system injuries. Cerebrospinal fluid, EEG and head MRI abnormalities also mainly occur in this group of patients. While this difference is not statistically significant, the limited sample size is probably to blame. As mentioned above, the disease progress rapidly, and apparent nervous system injury was observed at approximately 3 days. Body temperature rises to > 39.1°C accompanied by convulsions, coma, limb movement disorder and severe complications such as neurogenic shock or neurogenic pulmonary edema. For patients with no rash, EV71 infection should be strongly suspected (Tables 2, 3).

## Conclusion

The risk factors of life-threatening cases in hospitalized HFMD patients include young age, high fever, male gender and EV71 infection. HFMD patients infected with EV71 have a faster disease progression, higher fever and a higher incidence of limb movement disorder, coma, neurological damage, neurogenic pulmonary edema and death. Patients with neurological complications are prone to worse outcome and severe sequela. Generally, in HFMD patients, the symptoms of neurological damage caused by CA16 or other enteroviral infections are relatively mild, and the patients' prognoses are better. Severely ill patients, especially the critically and fatally ill patients are mostly infected by EV71; Nonetheless CA16 and other enteroviruses can also cause severe neurological symptoms in HFMD.

## Materials and methods

An outbreak of pediatric hand, foot and mouth disease occurred in Liaoning Province of China in 2010. Patients with severe symptoms were hospitalized, and those in grave condition were treated in the pediatric intensive care unit (PICU). A retrospective analysis of this outbreak was conducted using general clinical data and data on clinical manifestations. Our study has been approved by the institutional review board of China Medical University. Pathogen test were carried out after informing and gaining permission from the patients' parents. The study focused on neurological complications and findings related to the pathogens and their correlations with HFMD in patients admitted to Shengjing Hospital of China Medical University between January 1st and December 31st 2010.

### Grouping

The hospitalized patients were grouped by disease severity. A general patient was diagnosed with HFMD, and accompanied any one of the following symptoms: 1, lethargy, irritability or other behavioral signs of infection; 2, pneumonia and an increase in myocardial enzymes (particularly troponin, CPK-MB and their isozymes); 3, more than two incidences of vomiting accompanied by limb shaking;4, no improvement in symptoms 3 days after outpatient treatment or an unstable condition resulting in hospitalization. Severe group included patients presenting with obvious symptoms of nervous system involvement in HFMD, including complicating myoclonus, encephalitis, brain-stem encephalitis and acute flaccid paralysis in addition to the basic symptoms of HFMD. The critically ill patients were those whose treatment was complicated by central respiratory failure or those who had brain-stem encephalitis, acute flaccid paralysis, neurogenic pulmonary edema, neurogenic shock or other severe symptoms and required endotracheal intubation and mechanical ventilation. Fatal patients included patients who died of respiratory and cardiac arrest and those who suffered brain death during treatment.

A second grouping was based on the pathogens detected: group EV71, group CA16 and group OE referring to patients infected with other enteroviruses.

### Diagnosis

HFMD was defined as a febrile illness (temperature > 37.5°C) accompanied by a papulovesicular rash of the oral mucosa, limb extremities and/or buttocks. Encephalitis was defined as persistent fever, altered consciousness, vomiting, limb shaking and convulsions with or without changes as seen in tests of cerebrospinal fluid and head imaging. Brainstem encephalitis was defined as brain-stem injury accompanied by lethargy and cranial nerve palsy with the clinical manifestations of cerebellar ataxia in addition to encephalitis. AFP was defined as the acute onset of loss of motor function in at least 1 skeletal muscle group (usually the limbs) associated with absent or diminished reflexes in the affected muscle group(s). Neurogenic pulmonary edema was defined as having an acute onset with early difficulty in breathing accompanied by a large amount of bloody, frothy sputum, auscultation of the lungs, high blood pressure, apparent pulmonary edema detected by chest X-Ray or bloody sputum drawn into an endotracheal tube. The term dysfunction is used in reference to patients who, after acute-phase treatment, still had a decreased ability to swallow and cough as well as decreased body movement and intelligence or those who had stable vital signs but lacked regular and effective autonomous respiration and relied on prolonged mechanical ventilation. All diagnoses of nervous system disease were made with the support of a pediatric neurologist.

### Pathogen detection

Throat swab and/or stool specimens were collected from each child enrolled in the study. These samples were transported immediately at 4°C to the Centers for Disease Control and Prevention (CDC, Shenyang, China) for the detection of EV71, CA16 and OE using the enterovirus nucleic acid detection kit (triplex real-time polymerase chain reaction assay; Guangzhou Daan Gene Co., Ltd., Guangzhou, China).

Viral RNA was isolated with Trizol reagent (Invitrogen, Carlsbad, CA). The cDNA synthesis was performed using the RNA PCR Kit (AMV) Ver. 3.0 (TaKaRa, Dalian, China). The 25 μL PCR reaction mixture contained 1× buffer, 1.5 mM MgCl_2_, 0.2 mM dNTPs, 20 pmol forward primer and reverse primer and 20 pmol fluorescence-labeled probe for each target (see Table 4), 1 U of Taq polymerase and 5 μL of cDNA. Multiplex real-time PCR was performed on 7,500 Real-Time PCR System (Applied Biosystems, Foster City, CA) with the following conditions: 3 min at 94°C followed by 40 cycles at 93°C for 15 s and 55°C for 45 s.

**Table 4:** Nucleotide sequences of the specific primers and TaqMan probes used in this study

Primer (5'-3')		Probe (5'-3')
**pan-enterovirus**	Forward:GCAAGTCTGTGGCGGAACC Reverse:TGTCACCATAAGCAGCCATGATA	(FAM)- AATAACAGGAAACACGGACACCCAAAGTA(TAMRA)
**EV71**	Forward:GTTCACCTACATGCGCTTTGA Reverse:TGGAGCAATTGTGGGACAAC	(VIC)-TCTTGCGTGCACACCCACCG(TAMRA)
**CA16**	Forward:CCTAAAGACTAATGAGACCACCC Reverse:CTAAAGGCAGCACACAATTCG	(TEXAS RED)-CTTGTGCTTTCCAGTGTCGGTGCA(TAMRA)

The analysis of real-time PCR data: the threshold cycle (CT) value for each sample was calculated by determining the point at which the fluorescence exceeded the threshold limit, and the cut off was 35.1 determined by the ROC curve. The results of negative, positive and quality controls which were included in each experiment were within the normal range.

### Statistical analysis

Data analysis was conducted using the SPSS 13.0 software. Discrete variables are expressed as counts (percentages), and continuous variables are expressed as means ± standard deviation (SD). Differences in the demographic and clinical characteristics of the patient groups were assessed using the chi-square test for categorical variables. As the data showed a normal distribution, One-way ANOVA was adopted to analyze inter-subgroup differences. As the data did not show normal distribution, Kruskal Wallis Test was adopted to analyze inter-subgroup differences. *p *≤ 0.05 was considered statistically significant.

## Abbreviations

HFMD: Hand foot and mouth disease; EV71: Enterovirus71; CA: Coxsackie A; AFP: Acute flaccid paralysis; GBS: Gray-Barre syndrome; CRP: C reactive protein; PICU: Pediatric intensive care unit; CSF: Cerebrospinal fluid; EEG: Ambulatory eeg; MRI: Magnetic resonance imaging; HLA: Histocompatibility leukocyte antigen; GTARs: Gene-trap ankyrin repeats; SCARB2: Scavenger receptor class B Member 2.

## Competing interests

The authors declare that they have no competing interests.

## Authors' contributions

LC have made substantial contributions to conception and design; XW collected, analysed and interpreted the data and wrote the manuscript the data; YL and XX collected the data; WL, LJ and QY discussed and reviewed the manuscript; CR provided statistical analysis. All authors read and approved the final manuscript.
